# Aldolase A as a prognostic factor and mediator of progression via inducing epithelial–mesenchymal transition in gastric cancer

**DOI:** 10.1111/jcmm.13732

**Published:** 2018-07-11

**Authors:** Zhonghua Jiang, Xiaohong Wang, Jing Li, Hongmei Yang, Xin Lin

**Affiliations:** ^1^ Department of Gastroenterology The First People's Hospital of Yancheng Yancheng China; ^2^ Department of Gastroenterology The Second Affiliated Hospital of Xuzhou Medical University Xuzhou, Jiangsu China; ^3^ Departments of CyberKnife Huashan Hospital Fudan University Shanghai China; ^4^ Department of Digestive Endoscopy The First People's Hospital of Wujiang District Suzhou China

**Keywords:** ALDOA, epithelial–mesenchymal transition, gastric cancer, survival analysis

## Abstract

Glycolysis is regarded as the hallmark of cancer development and progression, which involves a multistep enzymatic reaction. This study aimed to explore the clinicopathological significance and potential role of glycolytic enzyme aldolase A (ALDOA) in the carcinogenesis and progression of gastric cancer (GC). ALDOA was screened from three paired liver metastasis tissues and primary GC tissues and further explored with clinical samples and in vitro studies. The ALDOA protein level significantly correlated with a larger tumor diameter (*P* = .004), advanced T stage (*P* < .001), N stage (*P* < .001) and lymphovascular invasion (*P* = .001). Moreover, the expression of ALDOA was an independent prognostic factor for the 5‐year overall survival and disease‐free survival of patients with GC in both univariate and multivariate survival analyses (*P* < .05). Silencing the expression of ALDOA in GC cell lines significantly impaired cell growth, proliferation and invasion ability (*P* < .05). Knockdown of the expression of ALDOA reversed the epithelial–mesenchymal transition process. Mechanically, ALDOA could affect the hypoxia‐inducible factor (HIF)‐1α activity as demonstrated by the HIF‐1α response element–luciferase activity in GC cells. Collectively, this study revealed that ALDOA was a potential biomarker of GC prognosis and was important in the carcinogenesis and progression of human GC.

## 1 INTRODUCTION

Gastric cancer (GC) is one of the most common and deadliest malignancies worldwide, especially in East Asia.^1^ Despite recent advancements in diagnosis and therapeutic methods, the prognosis of GC is still poor and the 5‐year survival rate for patients with GC has remained 20%‐25%^.^ 2, 3 The survival of patients largely depends on the disease stage at diagnosis. The tumour–node–metastasis staging system has been widely used as an effective approach to predict the prognosis of and determine the treatment option for GC. However, the challenges of considerable prognostic heterogeneity within each tumour stage need to be faced, as tumours at the same pathological stage can produce considerably different clinical outcomes,^4^, ^5^, ^6^, ^7^ highlighting the urgency for identification of other factors, such as the molecular mechanisms that influence metastases and prognosis of GC. Identifying biomarkers for early diagnosis and progression could help explain the biochemical mechanism underlying the processes of metastasis and progression as well as therapeutic sensitivity.

Tumour cells proliferate at a rapid rate and are always in a hypoxic microenvironment. Cancer cells develop adaptive responses to hypoxia by activating the hypoxia‐inducible genes to overcome this harsh environment.^8^, ^9^, ^10^ Under this circumstance, hypoxia is a major characteristic of cancer; in hypoxic conditions, cancer cells develop metabolism reprogram from oxidative phosphorylation to glycolysis.^11^, ^12^ A feature of this phenomenon is increased lactate production even at normal oxygen concentrations. This phenomenon is named as the Warburg effect or aerobic glycolysis. The Warburg effect was considered to be at the root of carcinogenesis and progression.^3^, ^13^, ^14^ It not only provides cancer cells with adenosine triphosphate (ATP) and nutrients but also leads to an acidic environment that facilitates metastasis.^3^ Glycolysis represents a 10‐step metabolic process that involves multiple enzymes.^15^ Studies have shown that some glycolytic enzymes are more complicated and multifaceted proteins than simple components of the glycolytic enzymes.^16^ These glycolytic enzymes have acquired additional non‐glycolytic functions in many aspects.^17^

In this study, quantitative reverse transcriptase–polymerase chain reaction (qRT‐PCR) arrays of glycolysis‐related enzymes were performed in three paired liver metastasis tissues and primary GC tissues. Aldolase A (ALDOA) was chosen for further study. This study mainly investigated the expression of ALDOA in GC tissues and its clinical significance and biological function in GC cells and also elucidated the mechanisms underlying the regulatory role of ALDOA in GC cells.

## 2 MATERIALS AND METHODS

### 2.1 Patients and samples

A series of 252 patients with pathologically confirmed GC was used for the immunohistochemical (IHC) study to explore the clinical significance of the expression of ALDOA in GC. All patients who had no neoadjuvant chemotherapy and received radical gastrectomy were randomly selected from samples collected between 2008 and 2012. The median follow‐up time was 46 months. The characteristics of the samples are shown in Table 1.

**Table 1:** Association between the expression of ALDOA and clinicopathological factors in gastric cancers

Characteristics	Total	ALDOA expression		*P* value
		Low expression	High expression	
		N (%)	N (%)	
Gender				
Male	134	44 (32.8)	90 (67.2)	.197
Female	118	30 (25.4)	88 (74.6)	
Age				
≥60	121	34 (30.5)	87 (69.5)	.672
<60	131	40 (28.1)	91 (71.9)	
Primary site				
Antrum/distal	95	26 (27.4)	69 (72.6)	.076
Cardia/proximal	86	28 (32.6)	58 (67.4)	
Fundus/body	48	18 (37.5)	30 (62.5)	
Gastroesophageal junction	23	2 (8.7)	21 (91.3)	
Diameter (cm)				
≥4	92	37 (40.2)	55 (59.8)	**.004**
<4	160	37 (23.1)	123 (76.9)	
Histologic grade				
G1/G2	108	38 (35.2)	70 (64.8)	.079
G3	144	36 (25.0)	108 (75.0)	
T stage				
T1/2	44	27 (61.4)	17 (38.6)	**<.001**
T3	111	24 (21.8)	86 (78.2)	
T4	101	23 (23.5)	75 (76.5)	
N stage				
N0	69	36 (52.1)	33 (47.8)	**<.001**
N1	52	10 (19.2)	42 (80.8)	
N2	58	9 (15.5)	49 (84.5)	
N3	73	19 (26.0)	54 (74.0)	
Lymphovascular invasion				
Negative	196	68 (34.7)	128 (65.3)	**.001**
Positive	56	6 (10.7)	50 (89.3)	
Perineural invasion				
Negative	185	58 (31.4)	127 (68.6)	.250
Positive	67	16 (23.9)	51 (76.1)	

Bold type indicates statistical significance.

This study was performed in compliance with the Helsinki Declaration. The use of clinical samples was approved by the Human Ethics Review Committee of the First People's Hospital of Yancheng, Yancheng, China. Written informed consent was obtained from all patients included in the study.

### 2.2 Cell lines

Human gastric adenocarcinoma cell lines, AGS and MGC‐803, were originally obtained from the Institute of Biochemistry and Cell Biology at the Chinese Academy of Sciences (Shanghai, China). The cells were cultured in the Dulbecco's modified Eagle's medium containing 10% foetal bovine serum, 100 U/mL penicillin and 100 μg/mL streptomycin in a 37°C incubator supplied with 5% CO_2_.

### 2.3 Western blot analysis

Protein extracts (50 mg) were resolved on 10% sodium dodecyl sulphate–polyacrylamide gel electrophoresis gels, transferred to nitrocellulose membranes (0.45 mm), and immunoblotted with rabbit anti‐human ALDOA antibody (11217‐1‐AP; 1:1000; Proteintech), anti‐E‐cadherin antibody (ab40772; 1:1000; Abcam), anti‐N‐cadherin antibody (ab18203; 1:1000; Abcam), anti‐Vimentin (ab92547; 1:1000; Abcam) or mouse anti‐human β‐actin monoclonal antibody (ab133626; 1:1000; Abcam). Images were developed using ECL (Pierce, Thermo Scientific, USA).

### 2.4 qPCR

The total RNA was extracted from cell lines using the TRIzol reagent (Invitrogen, CA, USA). qRT‐PCR was performed using the RNA PCR kit (TaKaRa, Dalian, China) and Fast SYBR Green qPCR Master Mix on Mx3000P (Stratagene, CA, USA) according to the manufacturer's protocol. The expression of β‐actin was used as an internal control. The primer sequence used was ALDOA‐F: ATGCCCTACCAATATCCAGCA and ALDOA‐R: GCTCCCAGTGGACTCATCTG.

### 2.5 IHC staining

Immunohistochemical staining was performed for ALDOA. Briefly, paraffin sections were baked for 60 minutes at 70°C, deparaffinized in xylene and rehydrated in gradually varied alcohol. Then, the sections were managed with 3% H_2_O_2_ to neutralize endogenous peroxidase for 30 minutes. The antigen retrieval was processed with citrate buffer (pH = 6.0) in a pressure cooker. After antigen retrieval, the sections were incubated with ALDOA primary antibody (11217‐1‐AP; 1:100; Proteintech) and secondary antibody. The sections were then stained with 3,3‐diaminobenzidine, terminated in phosphate‐buffered saline (PBS), and counterstained with haematoxylin. Based on the staining intensity of ALDOA in each case, the grading was as follows: 0, negative; 1, weak; 2, moderate; and 3, strong. The scores 0 and 1 were regarded as low expression and 2 and 3 as high expression. Two observers graded the score of staining intensity independently.

### 2.6 Stable transfection of GC cells

Biologically active short hairpin RNAs (shRNA) were generated using the lentiviral expression vector pLKO.1‐puro. The shRNA target sequence for human ALDOA was 5′‐CCATGCTTGCACTCAGAAGTT‐3′. PLKO.1‐scramble shRNA with limited homology having any known sequences in the human, mouse and rat genomes was used as a negative control. AGS and MGC803 cells were transfected with the pLKO.1‐shALDOA expression vector or pLKO.1‐scramble. The cells stably transfected were isolated using puromycin selection to obtain stable ALDOA knockdown cells.

### 2.7 Cell Counting Kit‐8 assays

The cells were seeded at a density of 2000 cells/well in 96‐well plates and incubated. An aliquot of 10 μL of Cell Counting Kit‐8 solution (CCK‐8; Dojindo, Kumamoto, Japan) was added to the wells and incubated for 2 hours. The absorbance was measured at 450 nm to calculate the numbers of viable cells in each well. Each measurement was performed in triplicate, and the experiments were repeated twice.

### 2.8 Colony formation assays

The cells were trypsinized and plated on six‐well plates (200 cells/well) and cultured for 2 weeks. The colonies were stained with 1% crystal violet for 30 seconds after fixation with 4% paraformaldehyde for 5 minutes. The colonies were counted and defined as >50 cells/colony. Three independent experiments were performed. The data were calculated using paired *t* test.

### 2.9 Transwell migration

Cell invasion assays were performed using 6.5‐mm Transwell chambers (8‐μm pore size, BD). The cells were seeded at a density of 50 000 cells/well into Transwell chambers coated with Matrigel for assays. The wells were washed with PBS after 36 hours of seeding. The cells that had migrated to the basal side of the membrane were fixed and stained with crystal violet, visualized and photographed using a CKX41 microscope (Olympus, Japan) at 200× magnification. Images of three random fields from three replicate wells were obtained, and the cells that had migrated were counted.

### 2.10 Statistical analysis

Comparisons between groups for statistical significance were performed with a two‐tailed paired Student's *t* test. The relationship between the expression of ALDOA and clinicopathological characteristics was tested using the chi‐square test. Survival curves were plotted by the Kaplan–Meier method and compared using the log‐rank test. The significance of various survival‐related variables was assessed using the Cox regression model in multivariate analysis. All statistical analyses were performed using the SPSS 17.0 statistical software package. A *P* value <.05 was considered statistically significant.

## 3 RESULTS

### 3.1 Glycolytic enzyme ALDOA might promote tumor metastasis in GC

**Figure 1:**
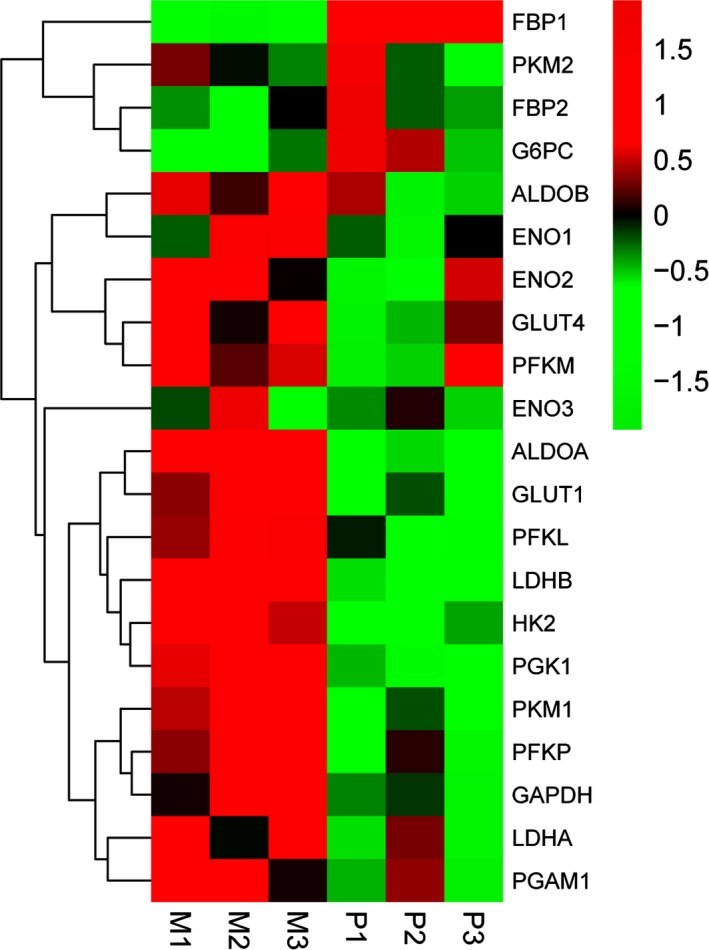
Heatmap of differentially expressed genes in three paired primary gastric cancer tissues and liver metastasis using qRT‐PCR. The results were normalized by *Z*‐score. Each column represented a specimen (denoted at the top), and each row represented a gene (denoted to the right). Red colour indicates genes that were up‐regulated, and green colour indicates genes that were down‐regulated. P represents primary gastric cancer tissue, and M represents corresponding liver metastasis

Glycolysis is a process involving a series of glycolytic enzymes. The qRT‐PCR assay was performed and the transcriptional expression levels of glycolytic enzymes in three paired GC tissues, and their paired synchronous liver metastasis tissues were measured to identify the glycolytic enzymes involved in gastric cancer metastasis. All patients underwent synchronous primary tumour and liver metastases resection. The results indicated that the up‐regulated expression of ALDOA and down‐regulated FBP1 were most significant in liver metastases compared with its primary tumour (Figure 1). As FBP1 has been studied previously,^18^ ALDOA was chosen for further study.

### 3.2 ALDOA was overexpressed in GC tissues

**Figure 2:**
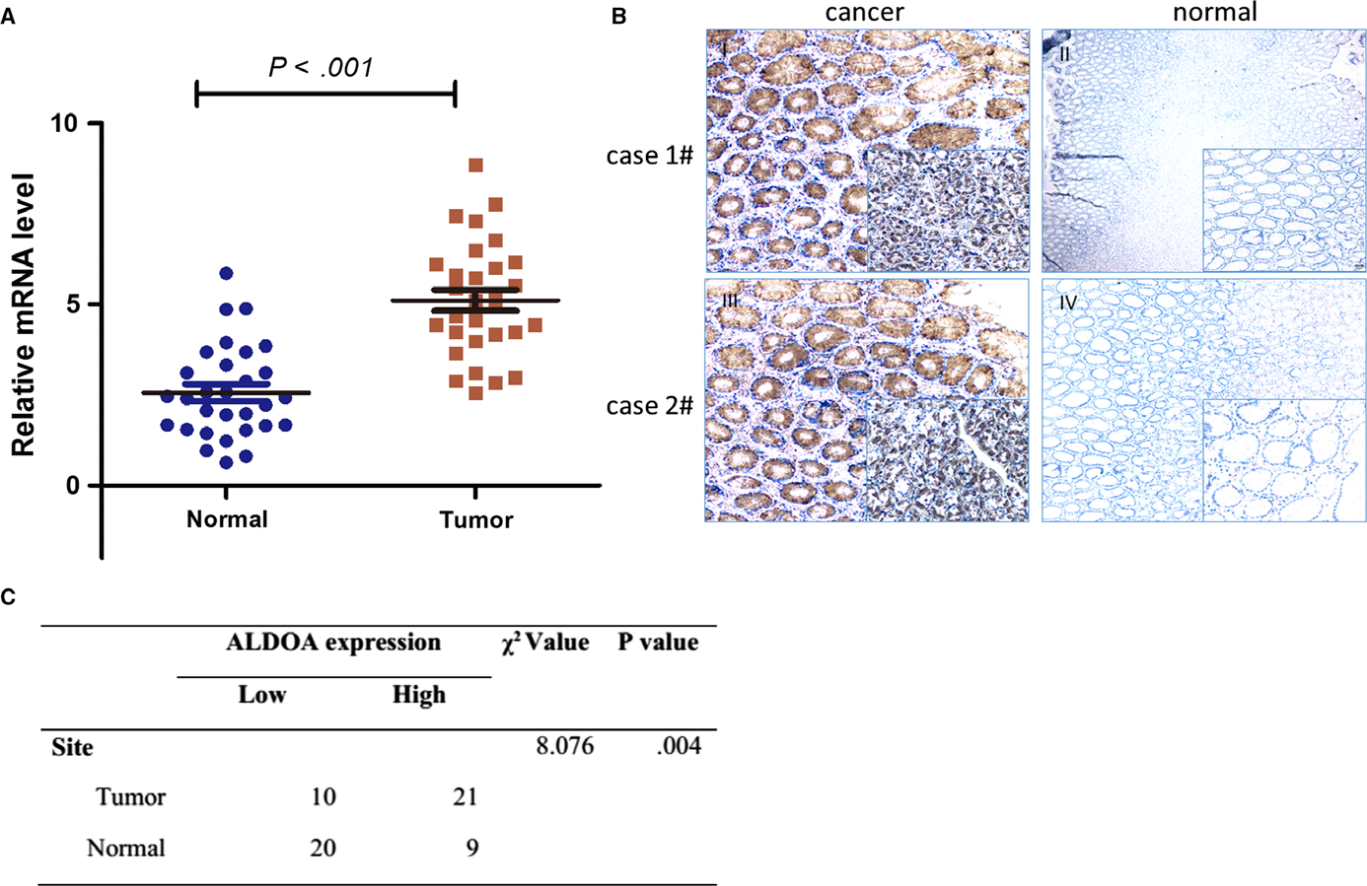
Aldolase A (ALDOA) was up‐regulated in gastric cancer (GC). A, The mRNA expression of ALDOA was found to be significantly up‐regulated in GC tissues than in adjacent normal controls in 30 patients with GC (*P* < .001). B, Examples of immunohistochemical (IHC) staining of the expression of ALDOA in GC (I and III) and normal gastric tissues (II and IV). C, The expression of ALDOA was significantly higher in GC tissues than in their adjacent normal control as determined by IHC staining (χ^2^ = 8.076, *P* = .004)

Quantitative reverse transcriptase–polymerase chain reaction and IHC staining were performed on 30 pairs of GCs (T) and matched adjacent gastric tissues (N) to explore whether ALDOA was dysregulated in human GC. As shown in Figure 2A, the mRNA expression of ALDOA increased 4.42‐ to 6.94‐fold in the GC tissues compared with their matched adjacent cervical tissues. As expected, the protein expression of ALDOA was also markedly up‐regulated in the GC tissues as judged by IHC (Figure 2B,C).

### 3.3 Expression of ALDOA correlated with inferior clinicopathological parameters

Immunohistochemistry of a series of 252 patients with primary GC was performed, including 134 males and 118 females with a mean age of 59 years (range, 21‐85 years), to investigate the clinicopathological significance of the expression of ALDOA. Of the 252 specimens, the expression of ALDOA was mainly observed in the cytoplasm in the majority of gastric cancerous specimens, with strong expression in 178 (70.6%) patients and weak or negative expression in 74 (29.4%) patients. Associations between the clinicopathological factors and the expression of ALDOA are summarized in Table 1. The increased expression of ALDOA significantly correlated with the depth of tumour invasion (T stage, *P* < .001), lymph node metastasis (N stage, *P* < .001), lymphovascular invasion (*P* = .001) and tumour diameter (*P* = .004) (Table 1). The factors not significantly associated with the staining included age (*P* = .672), sex (*P* = .197), primary tumour site (*P* = .076) and perineural invasion (*P* = .250).

### 3.4 Expression of ALDOA significantly correlated with survival outcomes in GC

**Figure 3:**
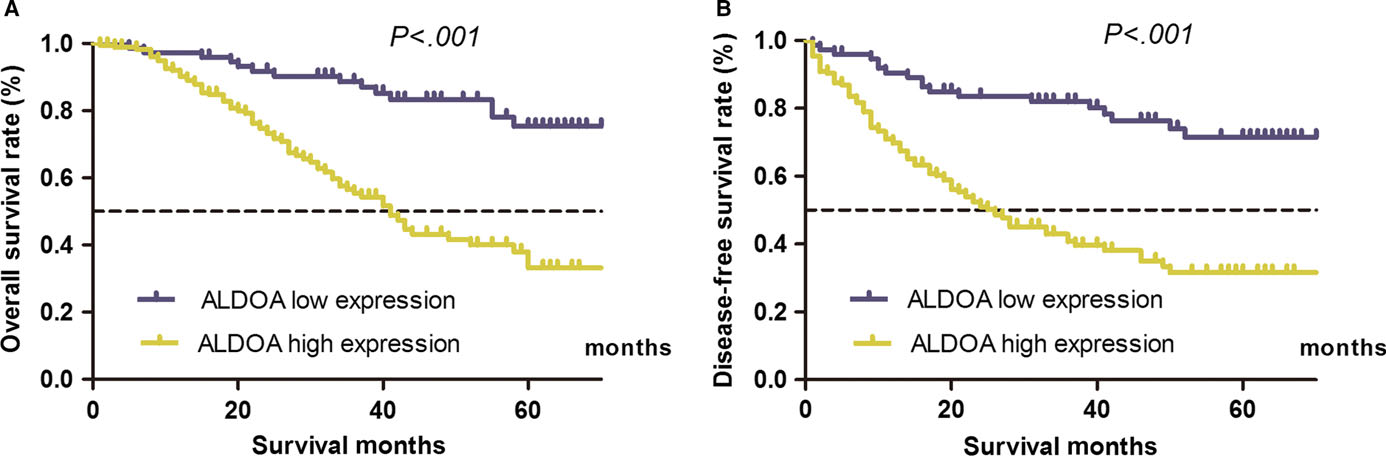
Strong expression of aldolase A (ALDOA) correlated with worse survival in patients with gastric cancer (GC). A, The 5‐y overall survival (OS) in the high‐ and low‐expression ALDOA groups was 39.2% and 76.9%, respectively (χ^2^ = 19.635, *P* < .001). B, The 5‐y disease‐free survival (DFS) in the high‐ and low‐expression ALDOA groups was 33.0% and 70.9%, respectively (χ^2^ = 28.857, *P* < .001)

This study further evaluated the relationship between the expression of ALDOA protein and the 5‐year overall survival (OS) or disease‐free survival (DFS) of patients. The results revealed a worse OS in patients with higher expression of ALDOA (high vs low expression: 39.2% vs 76.9%, χ^2^ = 19.635, *P* < .001) (Figure 3A). Similarly, a shorter DFS was also found in patients with a higher expression of ALDOA (high vs low expression: 33.0% vs 70.9%, χ^2^ = 28.857, *P* < .001) (Figure 3B). Moreover, multivariate survival analysis demonstrated that the expression of ALDOA was an independent prognostic factor for both OS (hazard ratio [HR], 2.596; 95% confidence interval [CI], 1.440‐4.680, *P* = .002) and DFS (HR, 2.824; 95% CI, 1.667‐4.783; *P* < .001) (Tables 2 and 3). The results of this study implicated that ALDOA might serve as a potential biomarker for survival in clinical practice.

**Table 2:** Univariate and multivariate Cox proportional hazards analyses of the expression of ALDOA gene and overall survival for patients with gastric cancer

Factor	Univariate analysis		Multivariate analysis	
	HR (95% CI)	*P*	HR (95% CI)	*P*
Gender	0.780 (0.514‐1.183)	.242		
Age	1.262 (0.832‐1.915)	.273		
Diameter	1.254 (0.810‐1.941)	.309		
T stage	2.029 (1.479‐2.782)	**<.001**	1.267 (0.824‐1.949)	.281
N stage	1.652 (1.365‐2.000)	**<.000**	1.442 (1.124‐1.850)	**.004**
Grade	1.694 (1.094‐2.625)	**.018**	1.179 (0.752‐1.848)	.474
Lymphovascular invasion	2.193 (1.404‐3.426)	**.001**	1.469 (0.923‐ 2.336)	.104
Perineural invasion	1.835 (1.193‐2.822)	**.006**	1.813 (1.163‐2.825)	**.009**
Tumor location	1.149 (0.957‐1.379)	.137		
ALDOA	3386 (1.909‐6.008)	**<.001**	2.596 (1.440‐4.680)	**.002**

CI, confidence interval; HR, hazard ratio.

Bold type indicates statistical significance.

**Table 3:** Univariate and multivariate Cox proportional hazards analyses of the gene expression of ALDOA and disease‐free survival for patients with gastric cancer

Factor	Univariate analysis		Multivariate analysis	
	HR (95% CI)	*P*	HR (95% CI)	*P*
Gender	0.949 (0.662‐1.361)	.775		
Age	1.132 (0.789‐1.624)	.500		
Diameter	1.187 (0.814‐1.729)	.373		
T category	2.068 (1.568‐2.728)	**<.001**	1.504 (1.056‐2.141)	**.024**
N stage	1.477 (1.260‐1.732)	**<.001**	1.218 (1.001‐1.483)	**.049**
Grade	1.917 (1.302‐2.822)	**.001**	1.447 (0.975‐2.149)	.067
Lymphovascular invasion	2.205 (1.497‐3.248)	**<.001**	1.489 (0.992‐2.235)	.055
Perineural invasion	1.503 (1.022‐2.211)	**.038**	1.388 (0.935‐2.061)	.104
Tumor location	1.069 (0.910‐1.257)	.416		
ALDOA	3.722 (2.221‐6.236)	**<.001**	2.824 (1.667‐4.783)	**<.001**

CI, confidence interval; HR, hazard ratio.

Bold type indicates statistical significance.

### 3.5 ALDOA knockdown inhibited proliferation and invasion of GC cells

**Figure 4:**
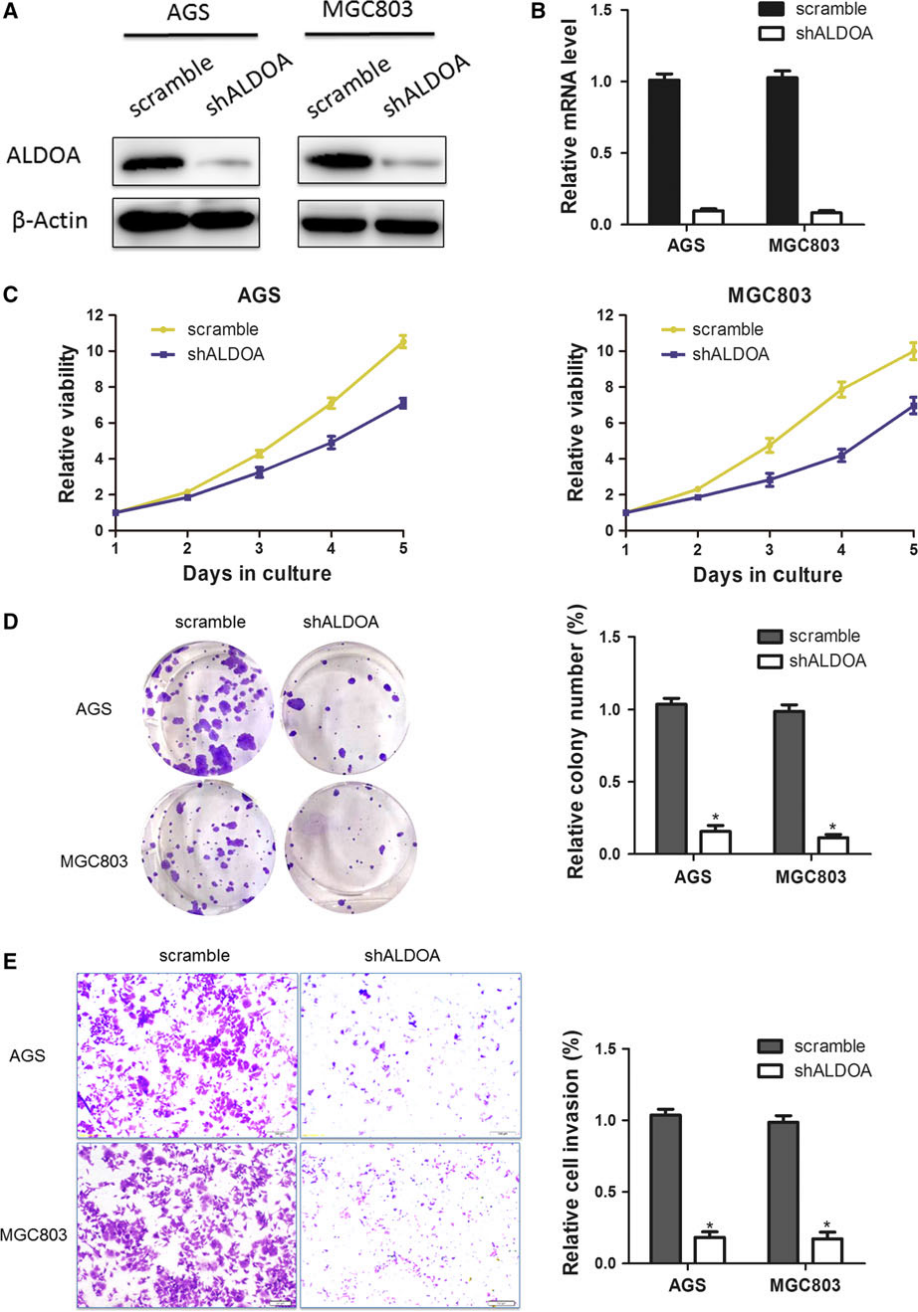
Knockdown of the expression of aldolase A (ALDOA) suppressed the proliferation and invasion abilities of gastric cancer cells. A, Western blot and (B) RT‐PCR analysis were performed to determine the knockdown effect of ALDOA in AGS and MGC803 cells. β‐Actin was used as the loading control. C, The growth curves of AGS and MGC803 cells with transfected shRNA–ALDOA or scramble sequence. Cell growth was determined using CCK‐8. D, A colony formation assay was performed to determine the oncogenic growth of AGS and MGC803 cells transfected with shRNA or scramble sequence. E, Transwell assays were performed to determine the invasion abilities of shRNA‐ALDOA‐transfected AGS and MGC803 cells. Values are presented as mean ± standard deviation of three independent experiments. *P < .05

Previous results indicated that ectopic ALDOA portended inferior clinical characteristics and shorter survival time of GC. Thus, it was hypothesized in this study that silencing the expression of ALDOA could inhibit growth and invasion of GC cells. The stable transfection models of the expression of ALDOA silencing in AGS and MGC803 cells were established. RT‐PCR and Western blot analysis were performed to verify that ALDOA transcriptional and protein levels decreased in both AGS and MGC803 cells after transfection with ALDOA–shRNA (Figure 4A,B). CCK‐8 and colony formation assays demonstrated that the proliferation of both AGS and MGC803 cells transfected with ALDOA–shRNA was significantly inactivated compared with that of those transfected with control shRNA (Figure 4C,D). Transwell assay showed that silencing the expression of ALDOA significantly inhibited the invasion of AGS and MGC803 cells **(**Figure 4E) (*P* < .05). These findings suggested that the knockdown of ALDOA repressed proliferation and invasion ability of GC cells.

### 3.6 Expression of altered ALDOA affects epithelial–mesenchymal transition induction in human GC cells

**Figure 5:**
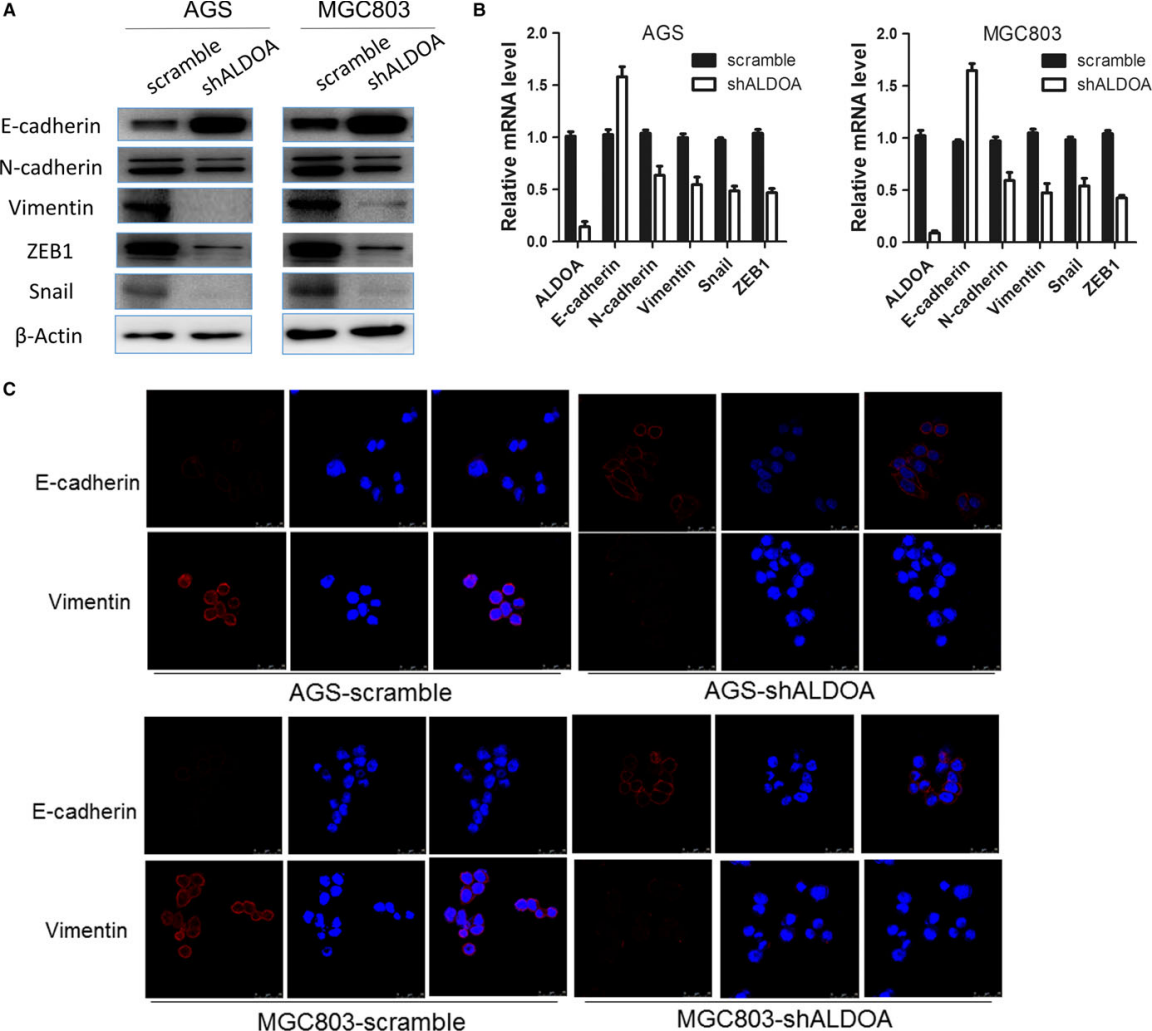
Silencing the expression of aldolase A (ALDOA) suppressed the epithelial–mesenchymal transition in gastric cancer. Silencing the expression of ALDOA resulted in the increased expression of E‐cadherin and decreased expression of vimentin, N‐cadherin, Snail and ZEB1, at both the protein (A) and transcriptional levels (B). C, Confocal microscopic analysis of phenotypic markers, including E‐cadherin, crystal violet and vimentin. The red signal represents the staining of corresponding protein and the blue signal represents the nuclear DNA staining using 4′,6‐diamidino‐2‐phenylindole

Thus, the expression of ALDOA was associated with GC metastasis and invasion ability. The capability of ALDOA to induce epithelial–mesenchymal transition (EMT) in human GC cells was subsequently investigated. The down‐regulated expression of ALDOA in AGS and MGC803 cells significantly decreased the levels of vimentin, N‐cadherin, Snail and ZEB1, but increased the expression of E‐cadherin at both protein and transcriptional levels (Figure 5A,B). Immunofluorescence staining further revealed that E‐cadherin was strongly induced while N‐cadherin decreased in ALDOA–shRNA‐transfected cells (Figure 5C). These results indicated that the altered expression of ALDOA affected EMT induction in GC cells.

### 3.7 ALDOA affected hypoxia‐inducible factor‐1α activity in GC

**Figure 6:**
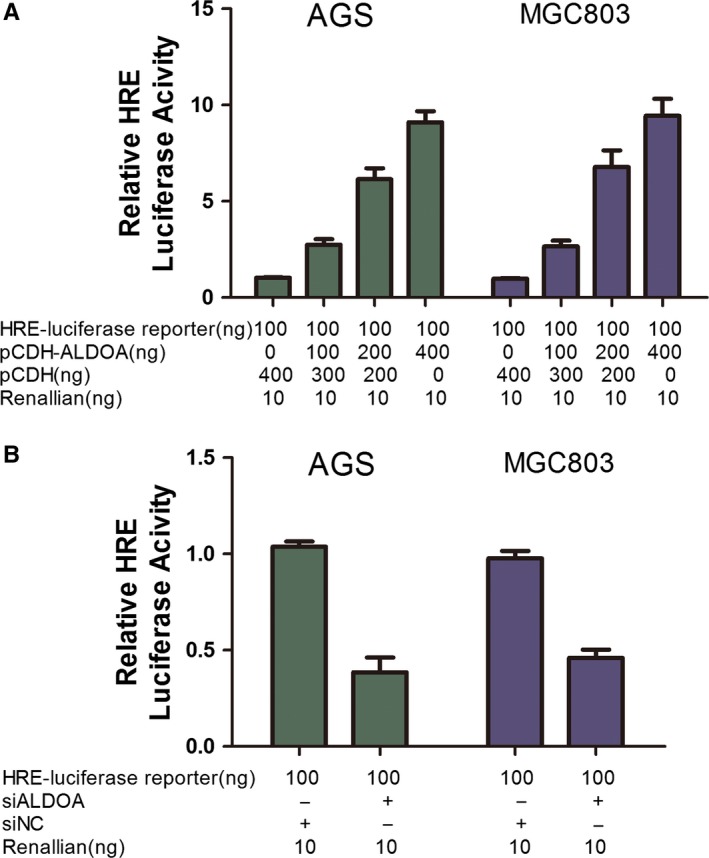
Aldolase A (ALDOA) affected the HIF‐1α activity in gastric cancer. A, It could increase the HRE‐luciferase activity in a dose‐dependent manner (*P* < .05). B, Silencing the expression of ALDOA decreased the HRE activity (*P* < .05)

Hypoxia‐inducible factor (HIF)‐1α is critical for tumour growth and metastases, in part by inducing glycolytic enzymes and EMT process. Glycolysis is also necessary for maintaining HIF‐1α activity. Therefore, it is possible that ALDOA can affect the HIF‐1α activity in GC. HIF‐1α acts by binding to the HIF‐1α response element (HRE) upon hypoxia. Therefore, this study used HRE‐luciferase reporter to examine whether ALDOA affected HRE‐luciferase activities. As expected, ALDOA could increase HRE‐luciferase activity in a dose‐dependent manner (Figure 6A). Moreover, HRE activity could be inhibited by reducing the expression of ALDOA using its specific shRNA (Figure 6B), implying that ALDOA indeed affected the HIF‐1α activity.

## 4 DISCUSSION

Great attention has been paid to glycolytic enzymes as potential therapeutic targets because the cancer‐specific metabolism depends more on the glycolytic pathway than on aerobic respiration, a phenomenon called the Warburg effect.^8^ This is a novel study that characterized the importance of ALDOA in GC. First, ALDOA was screened out by performing qRT‐PCR arrays of a panel of glycolysis‐related genes in three paired liver metastasis tissues and primary GC tissues. Then, the clinical significance and potential mechanisms by which ALDOA mediated GC metastases and progression were explored in detail. In fact, ALDOA has been validated as an oncogene in some solid tumours. In the pancreas, it promotes tumorigenesis and progression into a highly metastatic pancreatic cancer by regulating the expression of E‐cadherin.^19^ It regulates the cell cycle in non‐small cell lung cancer and several solid tumours.^20^

One of the most important findings in the present study was a direct relationship between the expression of ALDOA and the inferior clinicopathological features, and the high expression of ALDOA was an independent prognostic factor for both OS and DFS. The functional study indicated that silencing the expression of ALDOA impaired the proliferation and invasion abilities of GC cells. As EMT is the initial step of cancer metastases, the relationship between the expression of ALDOA and EMT‐related marker was further validated, which suggested that ALDOA might promote tumour metastases by inducing the EMT process.

HIF‐1α is critical for cancer cell survival and metastases in the solid tumour under stressed hypoxic tumour environment, in part by inducing glycolytic enzymes and EMT process.^21^, ^22^, ^23^ It could up‐regulate a series of glycolytic genes during anaerobic glycolysis and then increase the glycolysis rate and ATP production. A previous study also suggested that glycolysis was necessary for maintaining HIF‐1α activity.^24^ In the present study, the glycolytic enzyme ALDOA was also found to increase HIF‐1α activity. Glycolysis and HIF‐1α formed a feed‐forward loop that stimulated tumour growth and metastases. When silencing the expression of ALDOA, the anaerobic glycolysis was inhibited, ATP levels were reduced, the feed‐forward loop was broken and tumour proliferation and metastases were inhibited. Conversely, the high expression of ALDOA could increase the HIF‐1α activity, and HIF‐1α could, in turn, promote glycolysis and EMT process. Hence, ALDOA may serve as a promising biomarker and target therapy for GC.

Several studies focused on the relationship between EMT and glycolysis. A previous study demonstrated that FBP1 was a negative regulator of EMT in GC.^18^ Loss of FBP1 by Snail‐mediated repression provided metabolic advantages in basal‐like breast cancer.^25^ The ectopic expression of aldolase B was associated with poor prognosis and promotes tumour progression by EMT in colorectal adenocarcinoma.^17^ Some oncogene and tumour suppressors had a crosstalk between EMT and glycolysis in cancer progression and metastases, such as HIF‐1α,^23^ c‐myc,^26^ FOXM1,^27^ Gas1,^28^ and so on. The present study suggested a novel function of ALDOA other than a glycolytic enzyme.

Collectively, this study provided firm evidence that ALDOA was crucial in GC by inducing the EMT pathway and affecting HIF‐1α activity in tumour progression and metastasis. The expression level of ALDOA was an independent adverse prognostic factor for both OS and DFS, providing additional information for guiding therapeutic strategies.

## CONFLICTS OF INTEREST

None.
